# Investigation of the prevalence of Tn*916*/Tn*916*-like elements in oral streptococci

**DOI:** 10.1080/20002297.2017.1325273

**Published:** 2017-06-12

**Authors:** Tracy Lunde

**Affiliations:** ^a^ Department of Clinical Dentistry, Faculty of Health Sciences, UiT The Arctic University of Norway, Tromsø, Norway

## Abstract

*Streptococcus* species are the most abundant genera in the oral microbiota. The prevalence of antibiotic resistant oral streptococci is on the increase and the most reported antibiotic resistance profile in oral streptococci include, among several antibiotics, resistance to β-lactams antibiotics, macrolides and tetracycline. Several studies have shown that majority of the antibiotic resistance genes that have been isolated in oral streptococci are usually associated with mobile genetic elements such as plasmids and conjugative transposons. In the current study, we used molecular based techniques to screen a total of 100 clinical oral streptococci strains isolated in Norway for the presence of Tn*916*/Tn916-like elements. We also investigated the diversity of these elements in oral streptococci by Restriction Fragment Length Polymorphism (RFLP) and DNA sequencing. It was found that a total of 34 strains were positive for tetracycline resistance gene, *tet(M)*, and integrase gene *(int)* belonging to the Tn*916*-like family. The minimum inhibitory concentration (MIC) of these tetracycline-resistant oral streptococci ranged between 2-64 µg/mL. Most of the positive strains for *tet(M)* and *int* genes harbor the wild type Tn*916* and a few harbor other Tn*916*-like elements suggesting high versatility of the Tn*916* element in oral streptococci in Norway.

